# The complete mitochondrial genome of the hybrid species *Pungtungia herzi* (♀) ×* Pseudopungtungia nigra* (♂) from Korea

**DOI:** 10.1080/23802359.2021.1891980

**Published:** 2021-03-18

**Authors:** Kang-Rae Kim, Yeong-Ho Kwak, Mu- Sung Sung, Duc Tam Huynh, In-Chul Bang

**Affiliations:** a Department of Life Science & Biotechnology, Soonchunhyang University, Asan, Republic of Korea; b Inland Fisheries Research Institute, National Institute of Fisheries Science, Gapyeong, Republic of Korea

**Keywords:** Complete mitogenome, *Pungtungia herzi*, *Pseudopungtungia nigra*, hybrid

## Abstract

This study reports the complete mitochondrial genome of a *Pungtungia herzi* (♀) × *Pseudopungtungia nigra* (♂) hybrid. The *P*. *herzi* (♀) × *P*. *nigra* (♂) mitochondrial genome consists of 16,601 bp with 13 protein-coding genes, 2 ribosomal RNA genes, 22 transfer RNA genes, and a control region (D-loop). The overall base composition of the complete mitochondrial genome is 29.99% A, 26.97% T, 17.11% G, and 25.92% C. In the phylogenetic tree, *P*. *herzi* (♀) × *P*. *nigra* (♂) is closer to *P*. *herzi* than to *P*. *nigra*. Obtaining the complete mitogenome of *P*. *herzi* (♀) × *P*. *nigra* (♂) will provide useful data regarding *P*. *herzi and P*. *nigra* conservation and evolution.

Hybridization is common in fish and plays an important role in evolution (Hubbs 1955). *Pungtungia herzi* × *Pseudopungtungia nigra* natural hybrids have been reported in Ungcheoncheon Stream (Kim et al. 1991). *Pungtungia herzi* Herzenstein, 1892 and *Pseudopungtungia nigra* Mori, 1935 are species of Gobioninae and Cyprinidae, respectively. On the Korean Peninsula, *P*. *herzi* can be found in all rivers and streams, except in Hamgyeongbuk-do, and also occurs in northern China and southern Japan (Kim 1997). *Pungtungia herzi* has two reproductive strategies: obligate brood parasitism and crevice spawning (Yamane et al. 2009). *Pseudopungtungia nigra*, which is endangered due to habitat destruction and overfishing, is found only in Ungcheoncheon Stream and the Geumgang and Mangyeonggang rivers in South Korea, and its distribution is gradually decreasing. This species breeds only via obligate brood parasitism (Kim et al. 2004). *Pungtungia herzi × P. nigra* natural hybrids occur when *P. herzi* and *P. nigra* females both lay their eggs under a stone in the spawning ground of *Coreoperca herzi;* hybrids occur when *P. herzi* and *P. nigra* males release sperm and fertilize the eggs of either species (Kim et al. 2004; Yamane et al. 2009). Hybridization drives the differentiation of new species, and understanding thereof elucidates speciation and evolution (Demarais et al. 1992; Scribner et al. 2000). Therefore, a novel marker is needed in studies of evolution and genetics to identify hybrid *P. herzi × P. nigra*; to this end, artificial breeding was performed. In this study, we report the first complete mitochondrial genome of *P*. *herzi* (♀) × *P*. *nigra* (♂), which will provide useful data for phylogenetic and evolutionary studies.

A *P*. *herzi* (♀) × *P*. *nigra* (♂) hybrid was generated by artificial breeding of female *P*. *herzi* and male *P*. *nigra* from Yudeungcheon Stream (36°16′N, 127°23′E). *P*. *herzi* (♀) × *P*. *nigra* (♂) genomic DNA was extracted from ventral fin samples using a Genomic DNA Prep Kit (Biofact, Korea). The genomic DNA was preserved in the specimen room of Soonchunhyang University (voucher no. SUC-25117), Korea. To sequence the complete mitogenome, a DNA library was prepared using an MGIEasy DNA Library Prep Kit (MGI, China), and sequenced using 150 bp paired-end reads on the MGISEQ-2000 platform (MGI, Shenzhen, China). The raw datas were washed using Cutadapt 1.9 (Martin 2011) and assembled using Geneious (ver. 11.0.3). The assembled sequence was annotated using the MITOS web server (Bernt et al. 2013). The complete mitogenome sequence of *P*. *herzi* (♀) × *P*. *nigra* (♂) was deposited in GenBank with accession number MT974502.

The complete mitochondrial genome of *P*. *herzi* (♀) ×* P*. *nigra* (♂) comprises 16,601 bp, with 13 protein-coding genes (PCGs), 2 ribosomal RNAs (rRNA), 22 transfer RNA (tRNA) genes, and a control region (D-loop). The overall base composition of the complete mitochondrial genome of *P*. *herzi* (♀) × *P*. *nigra* (♂) was 29.99% A, 26.97% T, 17.11% G, and 25.92% C.

A phylogenetic tree was constructed using PhyML 3.0 software (Guindon et al. 2010) and MrBayes 3.2.7 (Ronquist et al. 2012), based on 13 PCGs of Cyprinidae (Figure 1). In the phylogenetic tree, the *P*. *herzi* (♀) × *P*. *nigra* (♂) hybrid mitochondrial genome was closer to *P*. *herzi* than to *P. nigra*. However, the *P. herzi* (♀) × *P. nigra* (♂) hybrid did not cluster very closely with *P. herzi*. In more detail, an analysis of p-distances (KF006339 and LC519883) based on 13 PCG sequences showed 0.099 between the *P*. *herzi* (♀) × *P*. *nigra* (♂) hybrid and *Pungtungia herzi*, reflecting an interspecies difference (the value for *P*. *nigra* was 0.107). This may have been due to mitochondrial recombination or difference in sequences between *P. herzi* populations. Therefore, the p-distance analysis was repeated using the *CO1* sequence of another *P*. *herzi* registered in NCBI. In this reanalysis of *P*. *herzi* (♀) × *P*. *nigra* (♂) and the *CO1* genes of the genera *Pseudopungtungia* and *Pungtungia*, the p-distances between the hybrid and *P*. *herzi* from Yudeungcheon Stream (MW002654∼MW002656) were very similar, ranging from 0.001 to 0.004. Notably, *P*. *herzi* from Yudeungcheon Stream differed in p-distance from other *P*. *herzi* (Japan, LC519883; Nakdong River, Korea, KF006339) by 0.086 to 0.088. *P*. *nigra* had a value of 0.085. Therefore, a molecular phylogenetic analysis is needed according to the geographic habitat of *P*. *herzi*. The complete mitogenome of *P*. *herzi* (♀) × *P*. *nigra* (♂) will provide a better understanding of *P*. *nigra* and *P*. *herzi* species evolution and conservation.

**Figure 1.**
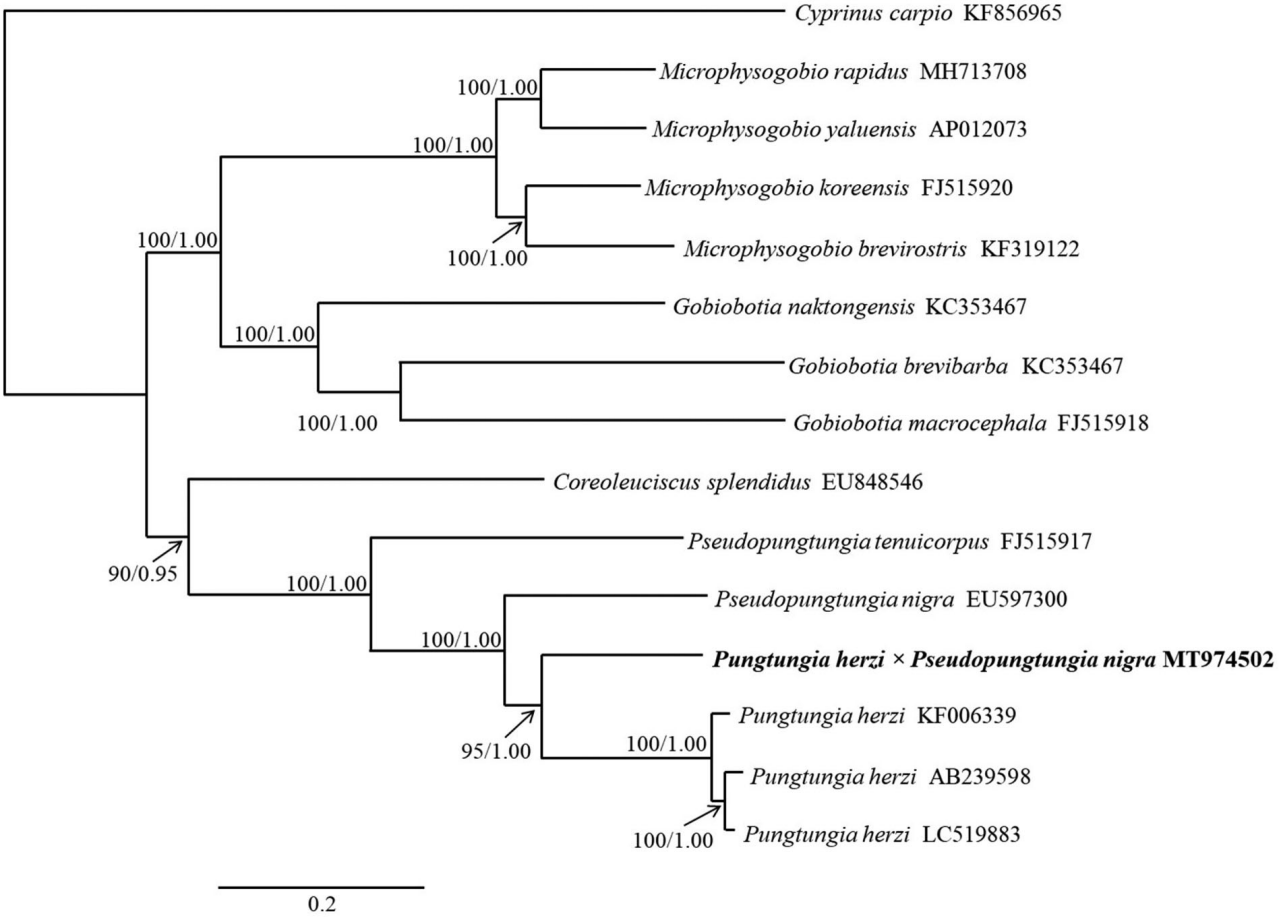
The phylogenetic tree constructed Bayesian inference and maximum likelihood based on 13 PCGs. The numbers above the nodes represent the bootstrap support value (left) and probability value (right) for each branch. The GenBank number for each species is indicated after the scientific name.

## Data Availability

The genome sequence data that support the findings of this study are openly available in GenBank of NCBI at (https://www.ncbi.nlm.nih.gov/) under the accession no. MT974502. The associated BioProject, SRA, and Bio-Sample numbers are PRJNA693092, SRX9902381 and SAMN17376903, respectively.
